# Accelerated 4D flow imaging using randomly undersampled echo planer imaging with compressed-sensing reconstruction

**DOI:** 10.1186/1532-429X-16-S1-W18

**Published:** 2014-01-16

**Authors:** Tamer A Basha, Kraig V Kissinger, Beth Goddu, Sophie Berg, Reza Nezafat

**Affiliations:** 1 Department of Medicine, Beth Israel Deaconess Medical Center, Boston, Massachusetts, USA

## Background

4D flow imaging using phase contrast CMR (PC CMR) allows visualization and quantification of blood flow. One of the major limitations of 4D flow imaging is its long scan time (in the order of 10-20 min). In this study, we sought to investigate an accelerate 4D flow imaging sequence that combines an efficient data sampling strategy using echo planar imaging (EPI) with randomly undersampled 3D k-space sampling pattern. The randomly undersampled k-space data are then reconstructed using compressed sensing (CS).

## Methods

**Figure 1:**
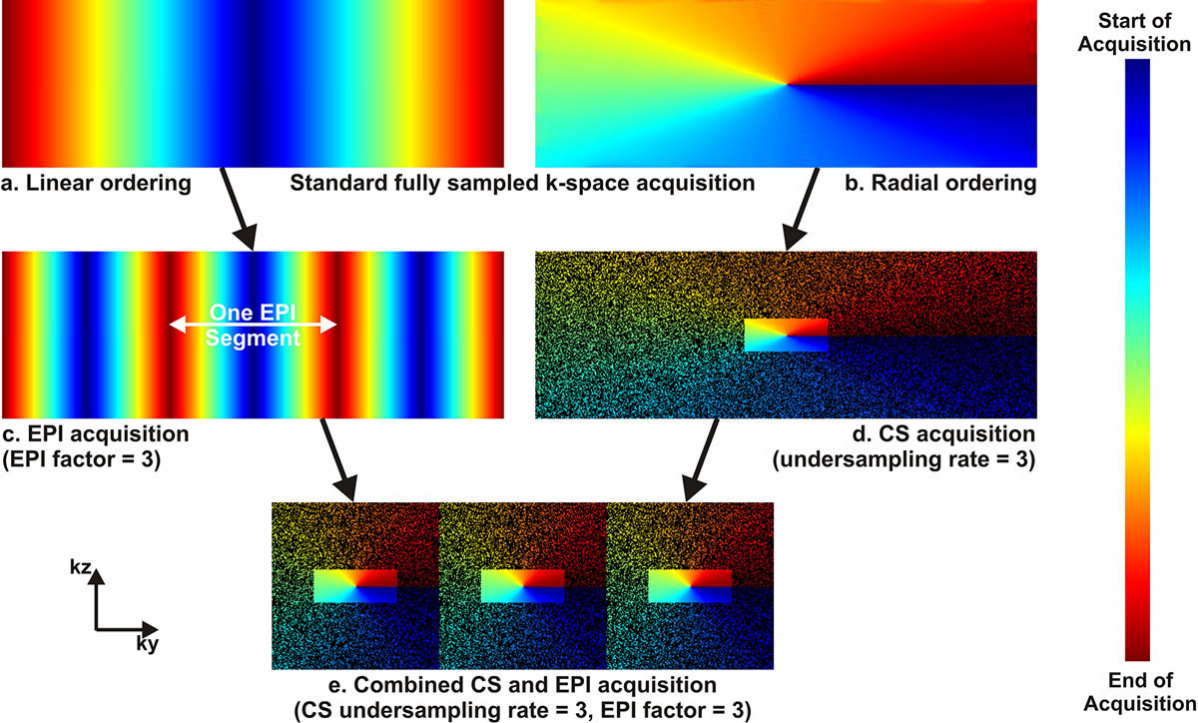
**K-space acquisition strategy: In regular 3D imaging, the profiles are acquired in either a linear (a) or radial ordering (b) fashion in the ky-kz plane**. Based on the linear ordering, EPI acceleration divides the k-space into multiple segments (c), where one line from each segment is acquired within the same EPI shot. On the other hand, CS acceleration is mostly based on the radial ordering (d), where the k-space profiles are randomly undersampled and acquired in a radial fashion while keeping the center area of the k-space fully sampled. Both EPI and CS can be combined into one acquisition with a higher acceleration rate as shown in (e). While the k-space is divided into segments, each segment is randomly undersampled with the same pattern and then acquired in a radial fashion. The major advantage is the high overall acceleration rate (9 in this ex.) for the whole 3D acquisition, while one drawback is the necessity to fully sample parts of the k-space even if it is not at the center of the k-space.

Figure 1 shows the k-space acquisition strategy. Similar to regular EPI acquisition, the k-space data are divided into multiple segments. For each segment, the profiles are undersampled with the CS rate such that all EPI segments have the same undersampling pattern. The proposed EPI random sampling strategy was implemented. Seven subjects were recruited (26 ± 12 years; 3 males) for 4D flow CMR on a 1.5T Philips Achieva magnet. Images were acquired axially using a GRE sequence (FOV = 340 × 280 × 60 mm^3^, resolution = 2 × 2 × 3 mm^3^, TR/TE/α = 7.4/3.8 ms/20°, EPI factor = 3, Turbo Factor = 2, CS rate = 3) in a volume covering the ascending and descending aorta, and the aortic bifurcation. Only foot-head flow encoding was used to provide an adequate temporal resolution of 30 ms for the measurements. A single beam navigator placed on the right hemi-diaphragm was used to gate the acquisition with the respiratory cycle. The nominal scan time for this scan was 3:30 minutes at 70 bpm assuming 100% gating efficiency (vs. 8:40 minutes if standard parallel imaging with rate 4 was used). For each subject, the 4D-PC scan was followed by a standard breath-hold 2D-PC scan with the same flow encoding direction (FOV = 340 × 280 mm2, resolution = 2 × 2 mm^2^, slice thickness = 5 mm^3^, SENSE rate = 2.5). The acquired 2D slice is selected from the previously obtained 4D scan and approximately at the aortic bifurcation. Data are then transferred to a separate station where the CS reconstruction was performed using a total variation minimization algorithm. Next, flow quantifications were performed on both the ascending and the descending aorta for all acquisitions and then compared between the 2D scans and the corresponding slices in the 4D scans.

## Results

**Figure 2:**
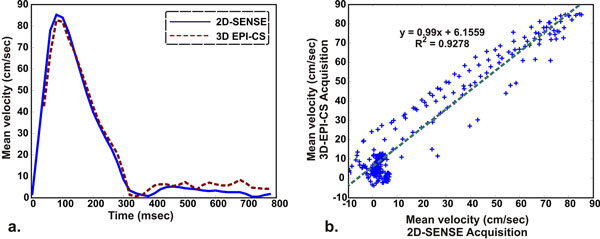
**a) Example mean velocity of the blood flow in ascending aorta from one subject using the 2D acquisition and the proposed 3D acquisition**. b) The correlation between the mean velocity measured from the 2D and the proposed 3D acquisitions.

Figure 2a shows representative mean velocity profiles for the ascending aorta of one volunteer using the 2D and 3D-EPI-CS acquisition. Figure 2b show the correlation between the mean velocity measurements from the 2D and the 3D-EPI-CS scans (R^2 ^= 0.93).

## Conclusions

A combination of EPI and randomly underampling k-space will substantially reduce the 4D flow scan time. Our initial results show no systematic difference between flow measurements between 2D PC CMR and 3D PC CMR.

## Funding

Samsung Electronics.

